# Clinical standards for the diagnosis and management of asthma in low- and middle-income countries

**DOI:** 10.5588/ijtld.23.0203

**Published:** 2023-09-01

**Authors:** S. Jayasooriya, M. Stolbrink, E. M. Khoo, I. T. Sunte, J. I. Awuru, M. Cohen, D. C. Lam, A. Spanevello, D. Visca, R. Centis, G. B. Migliori, A.C. Ayuk, J.A. Buendia, B. I. Awokola, B. E. Del-Rio-Navarro, S. Muteti-Fana, M. Lao-araya, P. Chiarella, H Badellino, S. W. Somwe, M. P. Anand, J. R. Garcí-Corzo, A. Bekele, M. E. Soto-Martinez, B. H. M. Ngahane, M. Florin, K. Voyi, K. Tabbah, B. Bakki, A. Alexander, B. L. Garba, E. M. Salvador, G. B. Fischer, A. G. Falade, Zorica Živković, S. J. Romero-Tapia, G. E. Erhabor, H. Zar, B. Gemicioglu, H. V. Brandão, X. Kurhasani, N. El-Sharif, V. Singh, J. C. Ranasinghe, S. T. Kudagammana, M. R. Masjedi, J. N. Velásquez, A. Jain, I. Cherrez-Ojeda, L. F. M. Valdeavellano, R. M. Gómez, E. Mesonjesi, B. M. Morfin-Maciel, A. E. Ndikum, G. B. Mukiibi, B. K. Reddy, O. Yusuf, S. Taright-Mahi, J. V. Mérida-Palacio, S. K. Kabra, E. Nkhama, N. R. Filho, V. B. Zhjegi, K. Mortimer, S. Rylance, R. R. Masekela

**Affiliations:** 1 Academic Unit of Primary Care, University of Sheffield, Sheffield; 2 Department of Clinical Sciences, Liverpool School of Tropical Medicine, Liverpool, UK; 3 Faculty of Medicine and Health Sciences, Stellenbosch University, Tygerberg, South Africa; 4 Faculty of Medicine, Universiti Malaya, Kuala Lumpur, Malaysia; 5 International Primary Care Respiratory Group, Edinburgh, Scotland, UK; 6 Global Allergy and Airways Patient Platform, Vienna, Austria; 7 Hospital Centro Médico, Guatemala City, Guatemala, Mexico; 8 Asociación Latinoamericana de Tórax, Montevideo, Uruguay; 9 Department of Medicine, University of Hong Kong, Hong Kong; 10 Asian Pacific Society of Respirology, Hong Kong, China; 11 Division of Pulmonary Rehabilitation, Istituti Clinici Scientifici Maugeri, Istituto di Ricovero e Cura a Carattere Scientifico, Tradate; 12 Department of Medicine and Surgery, Respiratory Diseases, University of Insubria, Varese-Como; 13 Servizio di Epidemiologia Clinica delle Malattie Respiratorie, Istituti Clinici Scientifici Maugeri, Tradate, Italy; 14 College of Medicine, University of Nigeria, Enugu, Nigeria; 15 Affiliation Departamento de Farmacologia y Tóxicologia, Facultad de Medicina, Universidad de Antioquia, Medellín, Colombia; 16 Medical Research Council, The Gambia at the London School of Tropical Medicine, The Gambia; 17 Hospital Infantil de México Federico Gômez, Mexico D.F, Mexico; 18 Department of Primary Care Sciences, University of Zimbabwe, Harare, Zimbabwe; 19 Division of Allergy and Clinical Immunology, Chian Mai University, Chiang Mai, Thailand; 20 Health Sciences School, Universidad Peruana de Ciencias Aplicadas, Lima, Peru; 21 Head Pediatric Respiratory Medicine Department, Clinica Regional del Este, San Francisco, Argentina; 22 Paediatrics and Child Health, University of Lusaka, Lusaka, Zambia; 23 Department of Respiratory Medicine, JSS Medical College, Mysore, India; 24 Department of Pediatrics, Universidad Industrial de Santander, Santander, Colombia; 25 College of Health Sciences, Addis Ababa University, Addis Ababa, Ethiopia; 26 Department of Pediatrics, Universidad de Costa Rica, San Jose, Costa Rica; 27 Douala General Hospital, University of Douala, Douala, Cameroon; 28 Institute of Pneumology M. Nasta, Bucharest, Romania; 29 School of Health Systems and Public Health, University of Pretoria, Pretoria, South Africa; 30 College of Medicine, Ajman University, Ajman, United Arab Emirates; 31 University of Maiduguri Teaching Hospital, Maiduguri; 32 Deparment of Medicine, University of Abuja, Abuja; 33 Department of Paediatrics, Usmanu Danfodiyo, University Teaching Hospital, Sokoto, Nigeria; 34 Deparment of Biological Sciences, Eduardo Mondlane University, Maputo, Mozambique; 35 University of Medical Sciences, Porto Alegre, RS, Brazil; 36 Department of Paediatrics, University of Ibadan, Ibadan, Nigeria; 37 Dragiša Mišovic, Childrens Hsopital for Lung Disease and TB, Belgrade, Serbia; 38 Health Sciences, Academic Division, Juarez Autononous, University of Tabasco, Villahermosa, Mexico; 39 Department of Medicine, Obafemi Awolowo University Teaching Hospital Complex, Ile-Ife, Nigeria; 40 Department of Paediatrics & Child Health & SA MRC Unit on Children & Adolescent Health, Red Cross Childrens Hospital, University of Cape Town, Cape Town, South Africa; 41 Department of Pulmonary Diseases, Istanbul University, Cerrahpasa, Turkey; 42 State University of Feira de Santana, Feira de Santana, BA, Brazil; 43 UBT Higher Education Institution, Prishtina, Kosovo; 44 Al-Quds University, Jerusalem, Palestine; 45 MJ Rajasthan Hospital, Jaipur, India; 46 Paediatrics Unit, Teaching Hospital Peradeniya, Kandy; 47 Faculty of Medicine, University of Peradeniya, Kandy, Sri Lanka; 48 Shahid Beheshti University of Medical Sciences, Tehran, Iran; 49 Medical School, Santander Industrial, Bucaramanga, Colombia; 50 Department of Community Medicine, Kasturba Medical College, Mangalore; 51 Universudad Espíritu Santo, Samborondón, Ecuador; 52 Francisco Morroguín University, Guatemala City, Guatemala; 53 Faculty of Health Sciences, Catholic University of Salta, Salta, Argentina; 54 Department of Allergy and Clinical Immunology, University Hospital Centre “Mother Teresa”, Tirana, Albania; 55 Hospital San Angel Inn, Mexico DF, Mexico; 56 The University of Yaounde 1, Yaounde, Cameroon; 57 Health Concern Initiative, Wakiso, Uganda; 58 Shishuka Children’s Speciality Hospital, Bangalore, India; 59 The Allergy and Asthma Institute, Islamabad, Pakistan; 60 Medecin Faculty, Mustapha Universitary Hospital Algiers, Algeria; 61 Centrode Investigación de Enfermedades Alérgicas y Respiratorias SC, Mexico DF, Mexico; 62 Pediatrics, All India Institute of Medical Sciences, New Delhi, India; 63 Levy Mwanawasa Medical University, School of Public Health and Environmental Sciences, Lusaka, Zambia; 64 Federal University of Parana, Curitiba, PA, Brazil; 65 Social Medicine, Medical Faculty, University of Prishtina, Prishtina, Kosovo; 66 University of Cambridge, Cambridge; 67 Imperial College, London; 68 Liverpool University Hospitals NHS Foundation Trust, Liverpool, UK; 69 Department of Paediatrics and Child Health, School of Clinical Medicine, University of KwaZulu Natal, Durban, South Africa; 70 Department of Non-communicable Diseases, World Health Organization, Geneva, Switzerland

**Keywords:** asthma, chronic respiratory disease, non-communicable disease, clinical standards, low-income and middle-income countries

## Abstract

**BACKGROUND:**

The aim of these clinical standards is to aid the diagnosis and management of asthma in low-resource settings in low- and middle-income countries (LMICs).

**METHODS:**

A panel of 52 experts in the field of asthma in LMICs participated in a two-stage Delphi process to establish and reach a consensus on the clinical standards.

**RESULTS:**

Eighteen clinical standards were defined: Standard 1, Every individual with symptoms and signs compatible with asthma should undergo a clinical assessment; Standard 2, In individuals (>6 years) with a clinical assessment supportive of a diagnosis of asthma, a hand-held spirometry measurement should be used to confirm variable expiratory airflow limitation by demonstrating an acute response to a bronchodilator; Standard 3, Pre- and post-bronchodilator spirometry should be performed in individuals (>6 years) to support diagnosis before treatment is commenced if there is diagnostic uncertainty; Standard 4, Individuals with an acute exacerbation of asthma and clinical signs of hypoxaemia or increased work of breathing should be given supplementary oxygen to maintain saturation at 94–98%; Standard 5, Inhaled short-acting beta-2 agonists (SABAs) should be used as an emergency reliever in individuals with asthma via an appropriate spacer device for metered-dose inhalers; Standard 6, Short-course oral corticosteroids should be administered in appropriate doses to individuals having moderate to severe acute asthma exacerbations (minimum 3–5 days); Standard 7, Individuals having a severe asthma exacerbation should receive emergency care, including oxygen therapy, systemic corticosteroids, inhaled bronchodilators (e.g., salbutamol with or without ipratropium bromide) and a single dose of intravenous magnesium sulphate should be considered; Standard 8, All individuals with asthma should receive education about asthma and a personalised action plan; Standard 9, Inhaled medications (excluding dry-powder devices) should be administered via an appropriate spacer device in both adults and children. Children aged 0–3 years will require the spacer to be coupled to a face mask; Standard 10, Children aged <5 years with asthma should receive a SABA as-needed at step 1 and an inhaled corticosteroid (ICS) to cover periods of wheezing due to respiratory viral infections, and SABA as-needed and daily ICS from step 2 upwards; Standard 11, Children aged 6–11 years with asthma should receive an ICS taken whenever an inhaled SABA is used; Standard 12, All adolescents aged 12–18 years and adults with asthma should receive a combination inhaler (ICS and rapid onset of action long-acting beta-agonist [LABA] such as budesonide-formoterol), where available, to be used either as-needed (for mild asthma) or as both maintenance and reliever therapy, for moderate to severe asthma; Standard 13, Inhaled SABA alone for the management of patients aged >12 years is not recommended as it is associated with increased risk of morbidity and mortality. It should only be used where there is no access to ICS.

The following standards (14–18) are for settings where there is no access to inhaled medicines. Standard 14, Patients without access to corticosteroids should be provided with a single short course of emergency oral prednisolone; Standard 15, Oral SABA for symptomatic relief should be used only if no inhaled SABA is available. Adjust to the individual’s lowest beneficial dose to minimise adverse effects; Standard 16, Oral leukotriene receptor antagonists (LTRA) can be used as a preventive medication and is preferable to the use of long-term oral systemic corticosteroids; Standard 17, In exceptional circumstances, when there is a high risk of mortality from exacerbations, low-dose oral prednisolone daily or on alternate days may be considered on a case-by-case basis; Standard 18. Oral theophylline should be restricted for use in situations where it is the only bronchodilator treatment option available.

**CONCLUSION:**

These first consensus-based clinical standards for asthma management in LMICs are intended to help clinicians provide the most effective care for people in resource-limited settings.

## Abstract (fr)

**CONTEXTE:**

L’objectif de ces normes cliniques est de faciliter le diagnostic et la prise en charge de l’asthme dans les environnements à faibles ressources des pays à revenu faible et intermédiaire (PRFI).

**MÉTHODES:**

Un groupe de 52 experts dans le domaine de l’asthme dans les PRFI a participé à un processus de Delphi en deux étapes afin d’établir et d’aboutir à un consensus sur les normes cliniques.

**RÉSULTATS:**

Dix-huit normes cliniques ont été définies : Norme 1, Toute personne présentant des symptômes et des signes compatibles avec l’asthme doit faire l’objet d’une évaluation clinique ; Norme 2, Chez les individus (>6 ans) avec une évaluation clinique soutenant un diagnostic d’asthme, une mesure de la spirométrie à main doit être utilisée pour confirmer une limitation variable du flux expiratoire en démontrant une réponse aiguë à un bronchodilatateur ; Norme 3, Une spirométrie pré- et post-bronchodilatateur doit être réalisée chez les individus (>6 ans) pour soutenir le diagnostic avant le début du traitement en cas d’incertitude diagnostique ; Norme 4, Les individus présentant une exacerbation aiguë de l’asthme et des signes cliniques d’hypoxémie ou de détresse respiratoire doivent recevoir de l’oxygène supplémentaire pour maintenir la saturation entre 94% et 98% ; Norme 5, Les bêta-2-agonistes à courte durée d’action (SABA) inhalés doivent être utilisés en tant que traitement d’urgence chez les individus asthmatiques via un dispositif d’inhalation adapté aux inhalateurs doseurs ; Norme 6, Les corticostéroïdes oraux à courte durée doivent être administrés à des doses appropriées aux individus souffrant d’une exacerbation aiguë modérée à sévère de l’asthme (minimum 3–5 jours) ; Norme 7, Les individus souffrant d’une exacerbation sévère de l’asthme doivent recevoir des soins d’urgence, y compris une oxygénothérapie, des corticostéroïdes systémiques, des bronchodilatateurs inhalés (par exemple, salbutamol avec ou sans bromure d’ipratropium) et une dose unique de sulfate de magnésium intraveineux doit être envisagée ; Norme 8, Tous les individus asthmatiques doivent recevoir une éducation sur l’asthme et un plan d’action personnalisé ; Norme 9, Les médicaments inhalés (à l’exception des dispositifs à poudre sèche) doivent être administrés via un dispositif d’inhalation adapté chez les adultes et les enfants. Les enfants de 0–3 ans auront besoin que le dispositif d’inhalation soit couplé à un masque facial ; Norme 10, Les enfants de moins de 5 ans asthmatiques devraient recevoir un bronchodilatateur à action rapide selon les besoins à l’étape 1, ainsi qu’un corticostéroïde inhalé (CSI) pour couvrir les périodes de respiration sifflante causée par des infections virales respiratoires. À partir de l’étape 2, ils devraient recevoir un bronchodilatateur à action rapide selon les besoins et un CSI quotidien ; Norme 11, Les enfants de 6 à 11 ans asthmatiques devraient recevoir un CSI chaque fois qu’ils utilisent un bronchodilatateur à action rapide inhalé ; Norme 12, Tous les adolescents de 12 à 18 ans et les adultes asthmatiques devraient recevoir un inhalateur combiné (CSI et bêta-agoniste à longue durée d’action [LABA] tel que le budésonide-formotérol ou le CSI/bronchodilatateur à action rapide), si disponible, à utiliser soit selon les besoins (pour l’asthme léger) ou en tant que traitement d’entretien et de secours pour l’asthme modéré à sévère ; Norme 13, L’utilisation d’un bronchodilatateur à action rapide inhalé seul pour la prise en charge des patients de plus de 12 ans n’est pas recommandée car elle est associée à un risque accru de morbidité et de mortalité. Elle devrait être utilisée uniquement en cas d’indisponibilité de CSI. Les normes suivantes (14–18) s’appliquent aux contextes où il n’y a pas d’accès aux médicaments inhalés ; Norme 14, Les patients qui n’ont pas accès aux corticostéroïdes devraient recevoir une seule cure courte de prednisolone oral en cas d’urgence ; Norme 15, Les bronchodilatateurs à action rapide par voie orale pour le soulagement des symptômes ne doivent être utilisés que s’il n’y a pas de bronchodilatateur à action rapide inhalé disponible. Ajuster à la dose minimale bénéfique pour minimiser les effets indésirables ; Norme 16, Les antagonistes des récepteurs des leucotriènes (LTRA) peuvent être utilisés comme médicaments préventifs et sont préférables à l’utilisation de corticostéroïdes systémiques oraux à long terme ; Norme 17, Dans des circonstances exceptionnelles, lorsque le risque de mortalité dû à des exacerbations est élevé, une dose faible de prednisolone oral quotidienne ou en jours alternés peut être envisagée au cas par cas ; Norme 18, La théophylline par voie orale devrait être réservée aux situations où c’est la seule option de traitement bronchodilatateur disponible.

**CONCLUSION:**

Ces premières normes cliniques basées sur un consensus pour la prise en charge de l’asthme dans les pays à revenu faible ou intermédiaire (LMIC) visent à aider les cliniciens à fournir les soins les plus efficaces aux personnes dans des environnements aux ressources limitées.

Asthma is a common respiratory disease affecting people of all ages and backgrounds, with considerable health and economic consequences.^1^–^5^ The majority of people with asthma can be managed effectively provided international asthma management recommendations (e.g., the Global Initiative for Asthma [GINA] strategy) can be implemented.^6^ Unfortunately, this is often not the case in low- and middle-income countries (LMICs), where limited access to affordable, quality diagnostic tests and treatments restrict the provision of effective asthma care.^1^,^2^,^7^–^10^ The WHO Package of Essential Non-communicable (PEN) disease interventions for primary healthcare includes step-wise guidance for asthma management based on inhaled short-acting beta-2 agonist (SABA) and inhaled corticosteroids (ICS),^11^ which are on the WHO Model List of Essential Medicines.^12^ However, these essential medicines are not always available, or affordable, or used in resource-limited settings.^3^,^5^,^8^,^13^ There are gaps between what is described in the currently available asthma management recommendations and the reality of what is typically available and affordable in LMICs. We have therefore defined clinical standards tailored to the specific challenges of managing asthma in these settings.

## AIM OF THE CLINICAL STANDARDS

Although our aim is to offer suggestions for the diagnosis and management of asthma in LMIC settings, we are not endorsing poor standards of care in LMICs. Instead, we are responding to the severe constraints on the resources available by presenting pragmatic approaches to provide effective clinical care. This consensus-based document describes the following activities:

1. Identifying and diagnosing asthma in children aged <5 years and 6–11 years, adolescents aged 12–18 years and adults in LMICs, considering the availability of equipment and potential context-specific differential diagnoses (Standards 1–3).
2. Managing acute exacerbations of asthma in children (<5 years, 6–11 years), adolescents (12–18 years) and adults where only WHO listed essential inhaled medications may be available (Standards 4–7).
3. Long-term management of asthma in children (<5 years, 6–11 years), adolescents (12–18 years) and adults if only WHO listed essential inhaled medications are available (Standards 8–13).
4. Long-term management of asthma in children (<5 years, 6–11 years), adolescents (12–18 years) and adults if there is no access to WHO listed essential inhaled medicines (Standards 14–18).

## METHODS

A panel of experts in the field of asthma in LMICs were identified from the Global Asthma Network (GAN) and International Union Against Tuberculosis and Lung Disease (The Union). Of the 69 experts initially invited, 56 (81.1%) responded to the first round of the Delphi process and 52 (92.9%) of these to the second round, representing 31 countries (see Supplementary Table S1). All respondents (*n* = 69) were asked to comment via a Delphi process on several draft standards developed by a core team of 14 researchers. At the first Delphi round, agreement was already high, with a median value of 93.7% (interquartile range [IQR] 85.5–96.9) for all standards. Following the second round of the Delphi process, agreement rose to a median of 96.2% (IQR 94.0–98.5) for all standards. Based on this substantial agreement, a draft document was developed following two rounds of the Delphi process by the expert panel. The document underwent multiple rounds of revisions, and the final version was approved by consensus (100% agreement).

This document uses the GINA definition for asthma: ‘Asthma is a heterogeneous disease, usually characterised by chronic airway inflammation. It is defined by the history of respiratory symptoms, such as wheeze, shortness of breath, chest tightness and cough that vary over time and in intensity, together with variable expiratory airflow limitation.’^6^

## STANDARD 1

**Every individual with symptoms and signs compatible with asthma should undergo a clinical assessment.**

Diagnosing asthma is based on the history of characteristic respiratory symptoms (wheeze, chest tightness, shortness of breath, dry cough), usually, but not exclusively, starting in childhood with a family history of atopy, confirmed by expiratory airflow limitation. Symptoms tend to be worse at night or early morning, varying in intensity over time and triggered by infections, allergens, irritant exposures, exercise, changes in weather and emotion. Physical examination may be normal but if patients are symptomatic, the most frequent finding is expiratory wheeze on lung auscultation.^14^ In LMICs, access to lung function testing is limited, and when available, is underused.^15^ The diagnosis of asthma is often made on clinical grounds, despite the knowledge that asthma may be under or over diagnosed and therefore, under- or over treated using this strategy.^16^,^17^ If this assessment is not suggestive of asthma, alternative diagnoses appropriate to the individual’s age and the context should be considered. The differential diagnosis may include other respiratory diseases of LMICs, e.g., TB, non-cystic fibrosis bronchiectasis, parasitic and fungal lung diseases, as well as chronic obstructive pulmonary disease (COPD) due to smoking or biomass exposure, cardiac diseases, dysfunctional breathing (vocal cord dysfunction) and foreign body inhalation.

## STANDARD 2

**In individuals (<6 years) with a clinical assessment supportive of a diagnosis of asthma, a hand-held spirometry measurement should be used, if possible, to confirm variable expiratory airflow limitation by demonstrating an acute response to a bronchodilator.**

Asthma is a clinical diagnosis, based on a history of characteristic symptom patterns and evidence of variable expiratory airflow limitation in lung function measurements, ideally based on spirometry revealing an obstructive pattern with a reduced forced expiratory volume in 1 sec (FEV_1_) and FEV_1_/FVC (forced vital capacity) compared to predicted population lower limit of normal (fixed cut-off FEV_1_ <0.75 in adults and <0.80 in children or fixed ratio of FEV_1_/FVC of <0.70 where lower limits of normal are unavailable).^18^,^19^ Normal spirometry does not exclude asthma and may require repeat testing. Given the lack of availability of spirometry in many primary care settings in LMICs, GINA^6^ and WHO PEN^11^ propose a peak expiratory flow (PEF) rate measurement demonstrating either an acute response to a bronchodilator or within-day variability over any 2-week period as an acceptable alternative to confirm variable expiratory airflow limitation. If unable to perform testing, a therapeutic trial is warranted if the history is suggestive of asthma. The likelihood of a diagnosis of asthma increases if there is any one of the following outside of respiratory infections:

1. A ≥20% improvement in PEF 15 min after the administration of 200–400 mcg inhaled salbutamol;
2. Excessive variability in twice daily PEF over 2 weeks (>10% and 13% in adults and children, respectively);
3. A significant increase in lung function after 2–4 weeks of anti-inflammatory treatment (>20% in PEF from baseline).

The greater the variations, or the more occasions excess variation is seen, the more confident the diagnosis.^20^

## STANDARD 3

**Pre- and post-bronchodilator spirometry should be performed in individuals (>6 years) to support diagnosis before treatment is commenced in case of diagnostic uncertainty.**

As there is no single objective test to diagnose asthma, misdiagnosis is common.^21^ One factor that contributes to misdiagnosis is failing to confirm reversible airflow obstruction,^22^ which should be documented prior to treatment commencing, as variability decreases post-treatment. Bronchodilator responsiveness is defined as >10% change relative to an individual’s predicted FEV_1_ and FVC.^23^ In LMICs, pre- and post-bronchodilator spirometry should be performed to support the diagnosis if other methods mentioned previously fail to demonstrate variable expiratory airflow limitation, or if there is a lack of response to treatment in children and adults.^6^,^21^,^24^

## ACUTE MANAGEMENT

## STANDARD 4

**Individuals with an acute exacerbation of asthma and clinical signs of hypoxaemia or increased work of breathing should be given supplementary oxygen to maintain SpO_2 _94–98%.**

Oxygen is prescribed for patients with hypoxaemia and is a marker of increased risk of a poor outcome. There are risks associated with both hypoxaemia and hyperoxia, which underlies the importance of prescribing oxygen only if required, and to within a target oxygen saturation range.^25^,^26^ A titrated oxygen regime is recommended in the treatment of severe asthma, in which oxygen is administered only to patients with hypoxaemia at a dose that relieves hypoxaemia without causing hyperoxaemia.^26^ In acute asthma, it is recommended to maintain an oxygen saturation of 93–95% in adults and 94–98% in children aged 6–11 years.^6^,^27^

## STANDARD 5

**Inhaled SABA should be used as an emergency reliver in individuals with asthma via an appropriate spacer device for metered-dose inhalers.**

Individuals presenting with an acute asthma exacerbation should receive frequent inhaled SABA therapy. The most efficient and cost-effective delivery method is by pressurised metered-dose inhaler (pMDI) and spacer.^28^ In LMICs, the cost and availability of commercial spacers may hinder the use of pMDIs. A practical alternative is to create a spacer by adapting clean, used 500 ml plastic drink bottles.^29^ A Cochrane Review demonstrated no inferiority between home-made and commercial spacers.^30^ In exacerbations with life-threatening features, oxygen-driven nebulisers are recommended to reduce the risk of oxygen desaturation.^31^

## STANDARD 6

**Short-course oral corticosteroids should be administered in appropriate doses to individuals having moderate to severe acute asthma exacerbations (minimum 3–5 days).**

Administration of corticosteroids reduces mortality, relapses, hospital admissions and the need for SABA therapy. The earlier the administration, the better the outcome.^32^ The oral route is as effective as the parenteral route and faster, less invasive and cheaper.^33^,^34^ The recommended doses in adults are equivalent to 50 mg prednisolone daily or 200 mg hydrocortisone (in divided doses) for 5–7 days; in children prednisolone 1–2 mg/kg up to maximum 40 mg/day for 3–5 days is advised.^6^

## STANDARD 7

**Individuals having a severe asthma exacerbation should receive emergency care including oxygen therapy, systemic corticosteroids, inhaled bronchodilators (e.g., salbutamol with or without ipratropium bromide), and a single dose of intravenous magnesium sulphate should be considered.**

The addition of an inhaled short-acting anticholinergic, e.g., ipratropium bromide, to standard initial treatment with oxygen, systemic corticosteroids and inhaled SABA has been shown to significantly decrease hospitalisations in adults and children with severe asthma exacerbations.^35^–^37^ The addition of a single infusion of intravenous magnesium sulphate also reduces risk of hospitalisation in adults and children with acute severe asthma,^37^,^38^ although evidence in children is limited. Intubation and mechanical ventilatory support should be considered where available and clinically indicated. Intravenous aminophylline increases the risk of side effects (nausea and tremor in children, vomiting, arrhythmias and palpitations in adults) without significant clinical benefit.^39^,^40^

## LONG-TERM MANAGEMENT

## STANDARD 8

**All individuals with asthma should receive education about asthma and a personalised action plan.**

Asthma education is a key component of asthma guidelines. In children, asthma self-management education programmes have demonstrated positive impact on a wide range of outcome measures.^41^ In adults, programmes including patient education and self-management support improved asthma-specific quality of life, severity scores and lung function tests.^42^ Differences in outcome reporting and in programme composition limit comparison between trials and ability for meta-analysis. However, training programmes empowering people to adjust their medication using a written action plan seem more effective than other forms of asthma self-management.^35^ The action plan (written or pictorial) should include inhaler technique assessment and advice about when and where to seek emergency treatment.

## STANDARD 9

**Inhaled medications (excluding dry-powder devices) should be administered via an appropriate spacer device in both adults and children. Children aged 0–3 years will require the spacer to be coupled with a face mask.**

To optimally manage asthma in both the long-term and during acute exacerbations, it is essential for inhaled medicines to be accessible and delivered effectively using appropriate spacer devices correctly, specifically when using pMDIs. The use of an appropriate spacer device is key to providing optimal pharmacotherapy, permitting maximal therapeutic dose delivery of the respirable aerosol fractions of inhaled medicines.^43^ Where commercial spacers are unavailable/affordable, spacer devices can be fashioned from plastic drink bottles.^29^

## STANDARD 10

**Children aged <5 years with asthma should receive a SABA as needed at step 1 and an ICS to cover periods of wheezing due to respiratory viral infections, and a SABA as needed and daily ICS from step 2 upwards.**

The overall goals of management in children younger than 5 years are ensuring good control of symptoms, maintaining normal lung growth, retaining healthy activity levels and minimising the risk of exacerbations, while avoiding adverse effects of medication. When symptoms or wheeze is mild, often only occasionally triggered by upper respiratory tract infections, step 1 management with SABA as needed can be considered as providing relief of symptoms. As symptoms become more frequent or definitive for the clinical diagnosis of asthma, step 2 management with addition of regular daily ICS for at least 3 months to achieve good control is essential.^44^,^45^

## STANDARD 11

**Children aged 6–11 years with asthma should receive an ICS, taken whenever an inhaled SABA is used.**

In children 6–11 years of age, the recommendation is the use of an ICS from step 1 whenever a SABA is taken. In a multi-centre study in children aged 5–18 years, the use of combined beclomethasone and salbutamol when symptomatic was found to be comparable to daily ICS use, with salbutamol alone as a reliever in children with mild persistent asthma.^46^ In a recent pragmatic real-world trial in African-American children aged 6–17 years, the use of beclomethasone and salbutamol when symptomatic showed similar levels of asthma control to use of daily beclomethasone.^47^ SABA overuse has been well described in adolescents and adults, and is associated with an increased risk of asthma exacerbations, urgent healthcare utilisation and mortality.^48^,^49^ Where access to healthcare facilities is limited, children should be provided with an ICS and SABA to prevent worsening asthma control and exacerbations.

## STANDARD 12

**All adolescents aged 12–18 years and adults with asthma should receive a combination inhaler (ICS and rapid onset of action long-acting beta agonist [LABA] such as budesonide-formoterol), where available, to be used either as needed (for mild asthma) or as both maintenance and reliever therapy, for moderate to severe asthma.**

There is a large body of clinical trial evidence from pragmatic and randomised control trials on the efficacy of combination inhalers with rapid onset of action LABA (budesonide-formoterol) as needed. In mild asthma, it prevents exacerbations and improves symptom control compared to use of short-acting beta agonists alone.^50^–^52^ A recent systematic review and meta-analysis confirmed the efficacy of this approach in mild asthma.^53^ Budesonide-formoterol is used as a maintenance and reliever therapy (MART) in moderate and severe asthma to reduce exacerbations.^51^,^54^–^56^ In settings where budesonide-formoterol is not available, other rapid onset of action LABA or ICS-SABA combinations should be used for patients to benefit from an anti-inflammatory medication whilst taking a reliever. This approach has been shown to reduce exacerbations.^57^ If an ICS-LABA/SABA combination inhaler is unavailable, patients should receive a regular inhaled corticosteroid and a separate inhaled SABA. SABA should never be used alone but in combination with ICS, as this approach reduces mortality and exacerbations.^48^,^58^,^59^

## STANDARD 13

**Inhaled SABA alone for the management of patients aged >12 years is not recommended as it is associated with a risk of increased morbidity and mortality. It should only be used where there is no access to ICS.**

Regular use of SABA, even for 1–2 weeks, is associated with reduced bronchodilator effectiveness, increased allergic response and eosinophilia.^60^,^61^ This can lead to a vicious cycle encouraging the overuse of SABA associated with increased numbers of exacerbations and mortality.^48^,^62^ Globally, 96% of asthma deaths occur in LMICs, and ensuring the regular use of ICS has the potential to reduce this burden.^3^

Clinical standards 1–13 for asthma management in LMIC settings where WHO essential medicines are accessible are listed in Table 1.

**Table 1:** 

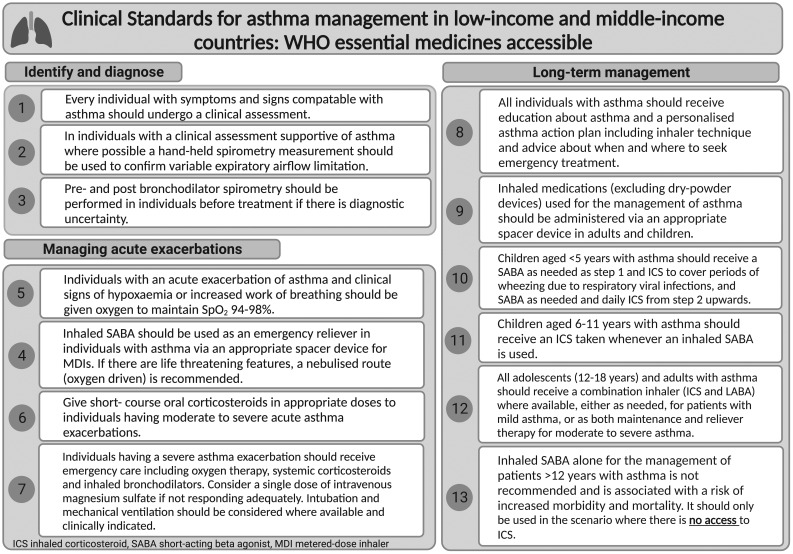

## SETTINGS THAT LACK INHALED MEDICATIONS

The following clinical standards for asthma are specific to LMICs and are not recommendations in current international guidelines. These are proposed for scenarios where inhaled medications are not available or affordable, especially ICS.^8^,^63^ This is not an endorsement of the primary use of oral medications for the treatment of asthma – wherever possible inhaled medication should be used as first-line pharmacological management as per all current international and national evidence-based asthma management guidelines. Inhaled medicines for asthma are considered best practice and should always be advocated at the primary care level. Oral salbutamol was removed from the WHO Model List of Essential Medicines in 2011. Oral SABA has a slow onset of action and higher doses are needed for bronchodilator effects similar to the inhaled form. In addition, higher rates of adverse effects such as tachycardia, hyperactivity, decreased oxygen saturation and tremors are seen. However, the reality remains that inhaled medications are not available in some LMICs.^64^

## STANDARD 14

**Patients without access to corticosteroids should be provided with a single short course of emergency oral prednisolone.**

Patients in remote/rural areas with limited access to corticosteroids should be provided with a single short course of emergency oral prednisolone to take only if needed as part of their asthma action plan. All patients should be provided with a written/pictorial action plan appropriate for their level of asthma control, so they know how to recognise and respond to worsening asthma.^65^,^66^ The dose of oral prednisolone (preferably given in the morning) is as per Standard 6, tapering is not needed if given for less than 2 weeks. In addition, we strongly recommend advocating to ascertain the timeline for the broader implementation of ICS treatment.

## STANDARD 15

**Oral SABA should be used for symptomatic relief only if no inhaled SABA is available. Adjust to the individual’s lowest beneficial dose to minimise adverse effects.**

The aim of asthma treatment is to improve respiratory symptoms, reduce inflammation and control acute exacerbations using a stepwise approach. Unfortunately, oral SABA is still used in about one in six adolescents and adults with poor asthma control in low-resource settings where access to alternative quality-assured essential asthma medicines is limited.^7^ Although no specific evidence or guidance is available, in the presence of previous or current adverse events it is useful to reduce the dose of oral SABA to the minimum effective dose, whenever alternative approaches are not available.

## STANDARD 16

**Oral leukotriene receptor antagonists (LTRA) can be used as a preventive medication and is preferable to the use of long-term oral systemic corticosteroids.**

Leukotrienes are inflammatory mediators to prevent and treat mild chronic asthma in association with ICS or as an alternative to ICS. These are less effective than ICS alone, and in 2020 the US Food and Drug Administration released a warning about the serious mental health adverse effects associated with montelukast,^67^ highlighting mood-related changes, including suicidal ideation and actions. Although in many patients these resolved on discontinuing treatment, they persisted in some patients with or without a history of prior mental illness. Where ICS is not available, as is the case in many LMICs, oral corticosteroid treatment may be effective in preventing future asthma exacerbations, but chronic use often leads to multiple adverse events.^68^ Oral LTRA may be used as an alternative medication and might be preferable to long-term oral systemic corticosteroids or oral salbutamol, but clinicians should consider the benefits and risks of mental health side effects before prescribing montelukast.

## STANDARD 17

**In exceptional circumstances when there is a high risk of mortality from exacerbations, low-dose oral prednisolone daily or on alternate days may be considered on a case-by-case basis.**

Low-dose oral prednisolone taken daily or on alternate days has been included in asthma treatment recommendations for several decades.^6^,^11^ This is typically a final step in recommendations after other asthma treatments with more favourable risk/benefit profiles have been considered. Given the historical context, this standard is included for settings where no inhaled therapies for asthma care are available. Low-dose oral prednisolone is an effective treatment for asthma addressing the underlying inflammatory nature of the disease and providing benefits in terms of symptom control and exacerbation risk reduction. However, in comparison to other asthma treatments, particularly ICS, it is associated with the risk of several dose-related adverse effects.^68^ Recognising and monitoring for side effects and titrating to an individual’s lowest beneficial dose is required. This standard is appropriate for carefully considered cases where on balance, the benefits outweigh the risks, and the lowest beneficial doses are used.

## STANDARD 18

**Oral theophylline should be restricted for use in situations where it is the onl*y* bronchodilator treatment option available.**

Oral theophylline has been included as an oral bronchodilator option in previous international asthma treatment recommendations. It has largely been superseded by other more effective treatments with more favourable side effect profiles. The narrow therapeutic window for theophylline makes its safe use difficult even in well-resourced healthcare settings, where drug levels can be measured and the dose titrated accordingly. Drug-level monitoring is often not an option in LMICs, making the safe use of theophylline even more challenging. When used in the absence of drug-level monitoring, we suggest not exceeding the lowest age-appropriate dose, accepting that this may result in sub-therapeutic dosing; however, this is preferable to potentially toxic dosing. We suggest that oral theophylline only be considered where there are no other oral (or inhaled) bronchodilator options. Oral SABA does not have a narrow therapeutic window in quite the same way, hence our preference for oral SABA over oral theophylline, although both have considerable systemic adverse effects compared to inhaled bronchodilators, which should always be used in preference wherever possible.

Clinical standards 14–18 for asthma management in LMIC settings that lack inhaled medications are listed in Table 2.

**Table 2:** 

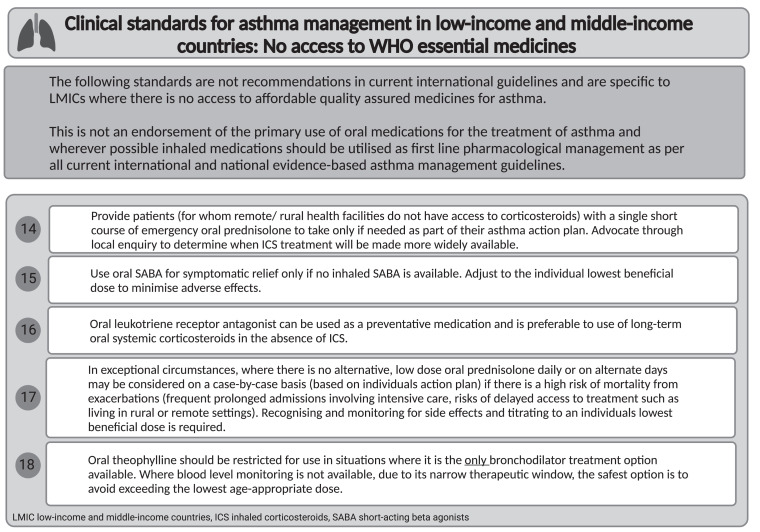

## CONCLUSION

The diagnosis of asthma requires a comprehensive clinical assessment, based on the history of characteristic respiratory symptoms and evidence of variable expiratory airflow limitation. Spirometry and PEF measurements are used to confirm variable expiratory airflow limitation, although in LMICs, where spirometry availability is limited, PEF measurements are commonly used. Maintenance treatment for asthma should include ICS; additional therapy can be added depending on the individual’s response to treatment. Effective management of asthma requires regular monitoring of symptoms and lung function, adherence to treatment and patient education to reduce exposure to environmental triggers.

These clinical standards are intended to assist clinicians with the difficult decisions they face in caring for people with asthma in resource-constrained settings. We also hope that they highlight the major global inequalities in access to basic effective and affordable asthma care. The WHO Global Action Plan for the Prevention and Control of Noncommunicable Diseases includes a target of 80% availability of the essential medicines to treat chronic respiratory diseases. Essential inhaled medicines must be part of universal health coverage to provide effective care for children, adolescents and adults with asthma wherever they live in the world.
