# Supplementary material for: Clinical standards for the diagnosis and management of asthma in low- and middle-income countries
Source: Int J Tuberc Lung Dis. 2023 Sep 1;27(9):658–67. doi: 10.5588/ijtld.23.0203 (PMC10443788; doi:10.5588/ijtld.23.0203)
Supplement: Supplementary file 1 [file iutld_ijtld_23.0203_supplementarydata1.pdf]

http://dx.doi.org/10.5588/ijtld.23.0203

# Clinical standards for the diagnosis and management of asthma in low- and middle-income countries

**Supplementary Table S1.** Low- and middle-income countries represented in the Delphi process.

| No. of Participants | WHO Region | Country | Income Group |
|---|---|---|---|
| 1 | Africa | Ethiopia | Low income |
| 1 | Africa | The Gambia | Low income |
| 1 | Africa | Mozambique | Low income |
| 1 | Africa | Uganda | Low income |
| 2 | Africa | Zambia | Lower middle income |
| 1 | Africa | Zimbabwe | Lower middle income |
| 2 | Africa | Cameroon | Lower middle income |
| 6 | Africa | Nigeria | Lower middle income |
| 1 | Africa | Algeria | Upper middle income |
| 2 | Africa | South Africa | Upper middle income |
| 1 | Eastern Mediterranean | Syrian Arab Republic | Low income |
| 1 | Eastern Mediterranean | Pakistan | Lower middle income |
| 1 | Eastern Mediterranean | Palestine | Lower middle income |
| 1 | Eastern Mediterranean | Iran | Upper middle income |
| 5 | South-East Asian | India | Lower middle income |
| 2 | South-East Asian | Sri Lanka | Lower middle income |
| 1 | South-East Asian | Thailand | Upper middle income |
| 2 | European | Kosovo | Lower middle income |
| 1 | European | Turkey | Upper middle income |
| 1 | European | Albania | Upper middle income |
| 1 | European | Romania | Upper middle income |
| 1 | European | Serbia | Upper middle income |
| 4 | Americas | Mexico | Upper middle income |
| 3 | Americas | Brazil | Upper middle income |
| 2 | Americas | Argentina | Upper middle income |
| 1 | Americas | Ecuador | Upper middle income |

| | | | |
|---|---|---|---|
| 3 | Americas | Colombia | Upper middle income |
| 1 | Americas | Peru | Upper middle income |
| 1 | Americas | Costa Rica | Upper middle income |
| 1 | Americas | Guatemala City | Upper middle income |
